# Complete genome sequence of *Bacillus cereus* NBNT-070, isolated from activated sludge of a wastewater treatment facility

**DOI:** 10.1128/mra.01168-25

**Published:** 2026-01-27

**Authors:** Yuxi Tian, Yong Min, Xiaoyan Liu, Yueying Wang, Yimin Qiu, Ben Rao, Ling Chen, Lei Zhu

**Affiliations:** 1 National Biopesticide Engineering Technology Research Center, Hubei Biopesticide Engineering Research Center, Biopesticide Branch of Hubei Innovation Centre of Agricultural Science and Technology, Hubei Academy of Agricultural Sciences, Wuhan, People's Republic of China; University of Southern California, Los Angeles, California, USA

**Keywords:** *Bacillus cereus*, activated sludge, wastewater treatment facility, short-read sequencing, long-read sequencing, genome, ecological role

## Abstract

*Bacillus cereus* is a widely distributed gram-positive bacterium that exhibits several notable characteristics. Here, we report the complete genome sequence of *B. cereus* strain NBNT-070, which was isolated from the activated sludge of a wastewater treatment facility. This data will be instrumental in elucidating the ecological role of *B. cereus*.

## ANNOUNCEMENT

*Bacillus cereus* is a large, gram-positive bacterium characterized by spore formation and peritrichous flagella (1). It exhibits considerable ecological versatility, inhabiting a wide range of environmental niches and demonstrating diverse lifestyle strategies (2). As an opportunistic pathogen implicated in foodborne illnesses, *B. cereus* is also used as a probiotic agent in both medical and animal husbandry contexts (3, 4). Moreover, certain strains promote plant growth and have been shown to exert biological control against various phytopathogens (5, 6). In this study, we report the genome sequence of *B. cereus* strain NBNT-070, which was isolated from activated sludge. The data will demonstrate the ecological functions and adaptive mechanisms of *B. cereus*.

Strain NBNT-070 was isolated from activated sludge collected from the wastewater treatment facility in Caidian District, Wuhan (30.52°N, 114.06°E), Hubei Province, China. Ten grams of activated sludge was placed in a triangular flask containing 90 mL sterile water and glass beads, then incubated at 30°C with 200 rpm shaking for 30 min. Then, the suspension was serially diluted and plated onto lysogeny broth (LB) agar media, which were incubated at 30°C for 24 h. Strain NBNT-070 was selected based on its distinct colony morphology on LB agar media and cultured in liquid LB overnight at 30°C with 220 rpm shaking to prepare biomass for genome sequencing.

Genomic DNA of strain NBNT-070 was extracted using the QIAGEN Genomic DNA Extraction Kit (QIAGEN, Germany). The DNA purity met the standard identified by a NanoDrop One UV-Vis Spectrophotometer (Thermo Fisher Scientific, USA), then it was split into two parts. One part was used for long-read sequencing, size-selected long DNA fragments were extracted using the PippinHT system (Sage Science, USA). The DNA library was constructed using the SQK-LSK114 library preparation kit (Oxford Nanopore Technologies [ONT], UK). Then, it was loaded onto the Nanopore PromethION sequencer (ONT). The data were generated and underwent quality control using Guppy in fast mode version 4.2.0 (7) which filtered out low-quality fail reads, resulting in 146,076 high-quality reads with an *N*_50_ length of 17,735 bps. The other part was used for short-read sequencing, genomic DNA was randomly sheared with a LE220 focused ultrasonicator (Covaris, USA), and DNA fragments with an average length of 200–400 bps were selected using MGIEasy DNA Clean beads (MGI, China). They then underwent end-repair, 3′ adenylation, adapter ligation, PCR amplification, and purification. The prepared libraries were sequenced on the DNBSEQ-T7RS platform (MGI, China) by paired-end PE150 sequencing. Quality control was performed using fastp version 0.23.1 (8), yielding a total of 16,549,274 reads comprising 2,470,501,072 bps. The two sequencing data sets were integrated to assemble a complete 5-replicon genome: 657× coverage (Unicycler v0.5.0) (9), 99.23% completeness and 0.15% contamination (CheckM v1.2.2) (10). The annotation of the genome was performed via NCBI PGAP version 6.10 (11), the details are shown in Table 1.

**TABLE 1:** Genome features of *Bacillus cereus* NBNT-070

Segment^*a*^	Length (bp)	G+C content (%)	No. of genes	No. of tRNAs	No. of rRNAs	Accession number
Chromosome	5,286,866	35.5	5,127	107	42	JBQVYV010000001.1
Plasmid 1	642,495	32.5	491	0	0	JBQVYV010000002.1
Plasmid 2	54,364	35.0	75	0	0	JBQVYV010000003.1
Plasmid 3	9,423	27.0	12	0	0	JBQVYV010000004.1
Plasmid 4	9,227	28.5	13	0	0	JBQVYV010000005.1
Total	6,002,375	35.00	5,718	107	42	JBQVYV000000000.1

^*a*^ The genomic sequence was aligned to the plasmid database within the NT database using blastn, and the length of the sequence that aligned to the database was calculated. A sequence is considered a plasmid if the aligned length accounts for more than 20% of the total sequence length and the total sequence length is less than 1 Mb.

Using an ANI calculator (12), the genome of strain NBNT-070 exhibited an average nucleotide identity of 98.62% compared to the *B. cereus* type strain ATCC 14579 (GenBank accession number CP034551.1). NBNT-070 was classified as a strain within the species *B. cereus*. All bioinformatics approaches were used with default parameters.

## Data Availability

The genome sequence of *Bacillus cereus* strain NBNT-070 has been deposited in the GenBank database under accession number JBQVYV000000000.1. The raw sequencing data were deposited in the Sequence Read Archive (SRA) database under accession numbers SRR35203685 and SRR35203686.
